# Adrenal insufficiency in the preterm infant

**DOI:** 10.1186/1824-7288-41-S1-A30

**Published:** 2015-09-24

**Authors:** Simonetta Picone, Roberto Aufieri, Piermichele Paolillo

**Affiliations:** 1 Division of Neonatology and Neonatal Intensive Care, Casilino General Hospital, Roma, Italy

## 

Cortisol production by the human fetal adrenal cortex has been shown to be not adequate at early gestation. This is due to an inefficient expression of 3β-hydroxysteroid dehydrogenase, the enzyme that catalyzes the synthesis of progesterone from pregnenolone, before about 23 weeks of gestation. Whereas, the fetal adrenal cortex it is able to produce dehydroepiandrosterone sulphate for placental estrogen synthesis and to convert placental progesterone to cortisol [1,2].

During the last trimester of pregnancy the fetal adrenal gland undergoes significant anatomical and functional maturation for adaptation to extra-uterine life and cortisol production increases greatly in the last two months of gestation. Cortisol increases synthesis of surfactant, enhances the reabsorption of lung fluid, promotes the conversion of T4 to T3, favors the closure of the ductus arteriosus and maturation of liver and intestinal enzymes [2].

Adrenal insufficiency can be caused by rare conditions such as adrenocortical hypoplasia or congenital enzymatic deficiencies of steroidogenesis, with clinical variables depending on the hormone involved.

In preterm infants, the developmental immaturity, combined with increased demands in critical illness, may result in insufficient cortisol production to maintain homeostasis in the face of acute stress or illness, despite apparently normal cortisol levels. This condition is known as “transient or relative adrenal insufficiency” (TAI) [3,4].

TAI usually appears in the first week of life and normalizes in the second week. Infants with TAI can exhibit refractory hypotension (hypotension not responsive to volume expanders and inotropic drugs, but responsive to corticosteroids), respiratory distress, patent ductus arteriosus and bronchopulmonary dysplasia (BPD) [2,3]. Diagnosis of TAI is not easy because many other conditions can cause hypotension in VLBW infants: hypovolemia, myocardial dysfunction, deficient vascular tone, hyaline membrane disease, infection or a combinations of these factors. In addition, normal basal cortisol levels are extremely variable in preterms and to date there is no consensus on the diagnosis of adrenal insufficiency based on tests with adrenocorticotropic hormone (ACTH) [5-8]. Appropriate dose and duration of steroid therapy have not been established yet. On the other hand refractory and persistent hypotension is associated with intraventricular hemorrhage, periventricular leukomalacia, increased mortality and neurological disability [9]. Poor adrenal response has been shown to be associated with later development of BPD and with death [10,11].

For these reasons further studies are needed to evaluate the efficacy and safety of glucocorticoids in the treatment of cardiovascular failure due to TAI in ill preterm and term infants [12,13].
